# Play Nicely: Evaluation of a Brief Intervention to Reduce Physical Punishment and the Beliefs That Justify It

**DOI:** 10.3390/children11050608

**Published:** 2024-05-20

**Authors:** Danna Valentina Nuñez-Talero, Martha Rocío González, Angela Trujillo

**Affiliations:** Facultad de Psicología y Ciencias del Comportamiento, Universidad de La Sabana, Chía 250005, Colombia; dannanunta@unisabana.edu.co (D.V.N.-T.); angela.trujillo@unisabana.edu.co (A.T.)

**Keywords:** intervention, brief, physical punishment, beliefs, efficacy

## Abstract

The objective of this study was to assess the efficacy of the Play Nicely brief intervention in diminishing both the utilization of physical punishment and the beliefs that endorse such behavior among a sample of Colombian parents with children aged 2 to 6. Utilizing a quasi-experimental design, the research included pretest and posttest evaluations and involved both an intervention group (*n* = 37) and a control group (*n* = 29). The assessment tools used were a scale to measure beliefs about the positive impacts of physical punishment and the Physical Assault subscale of the Spanish version of the Conflict Tactics Scale Parent–Child (CTSPC). Parents participated in a single online session, which offered eight interactive options and lasted 10 min. The results highlighted a high prevalence of physical punishment within the sample (81.8%) and established statistically significant correlations between the justification of physical punishment and its actual use. Approximately one month following the intervention, there was a significant reduction in the employment of physical punishment among the intervention group (*p* = 0.009), and a notable decrease in the belief that “Punishment is the best alternative to control children’s behavior” (*p* = 0.010) was observed. Consequently, the Play Nicely intervention proved effective in curtailing the use of physical punishment among parents of young children, demonstrating both efficacy and cost-effectiveness within a brief timeframe.

## 1. Introduction

Physical punishment is considered a violation of the major human rights treaties, such as the Universal Declaration of Human Rights and the Convention on the Rights of the Child [1,2,3,4]. In addition, Sustainable Development Goal (SDG) 16, Peace, Justice, and Strong Institutions, includes among its indicators reducing the proportion of children aged 1 to 17 years who have experienced any form of physical punishment by their caregivers in the last month [5].

Despite physical punishment being a violation of human rights and lacking any scientifically supported benefits for child development, it remains a prevalent disciplinary method among parents across various cultures [6,7]. According to the World Health Organization [8], approximately 300 million children aged 2 to 4 worldwide are subjected to violent forms of punishment. In Latin America and the Caribbean, UNICEF [9] reports that about one in two children experience physical punishment in their homes. Specifically, in Colombia, a study by Cuartas [10] revealed that around 1.7 million children under the age of five are exposed to physical punishment, indicating a prevalence rate of about 40%. Furthermore, a more recent investigation by Trujillo et al. [11] into the prevalence, chronicity, and severity of physical punishment involved a sample of 853 parents and their 1337 children, finding a remarkably high prevalence rate of 77%. These data underscore the widespread use and acceptance of physical punishment as a disciplinary practice, despite its adverse implications for children’s rights and well-being.

In 2021, Colombia enacted Law 2089 [12], which prohibits the use of physical punishment, cruel, humiliating, or degrading treatment, and any form of violence as a means of correction against minors. The law introduced various provisions to safeguard the rights of children and adolescents, such as the development of a national pedagogical strategy with the objective of “altering societal and cultural perceptions, beliefs, and behaviors that have historically normalized and rationalized physical punishment” [13] (p. 38). Numerous studies have demonstrated a positive correlation between the endorsement of physical punishment and the increased risk of physical abuse, particularly among parents who possess strong favorable views towards this negative disciplinary method [14,15,16,17,18]. In nations with prolonged histories of armed conflict, such as Colombia, there is evidence to suggest that the population may normalize violence, thereby creating an environment where parents might view punitive measures against their children as justified [19,20,21].

However, Colombia currently lacks comprehensive pedagogical strategies specifically tailored to reducing physical punishment, and many existing interventions have not been validated for the Colombian context. Consequently, there is a pressing need for evidence-based interventions that address and alter harmful parental beliefs, thereby reducing the incidence of physical punishment and its associated risks in Colombian society. Thus, the primary objective of the current study was to assess the efficacy of the Play Nicely brief intervention in mitigating instances of physical punishment and the underlying beliefs that support it among parents of children aged 2 to 6 years old.

### 1.1. Physical Punishment

Physical punishment is operationally defined as the deliberate application of physical force intended to induce physical discomfort without causing injury, with the aim of disciplining or regulating the behavior of a child [22]. The prevalence of physical punishment denotes the ratio of individuals employing such disciplinary measures compared to the total population under examination at a particular point in time [23]. The incidence of corporal punishment spans a spectrum of frequency, ranging from isolated incidents to regular occurrences as a routine aspect of parental disciplinary practices aimed at modifying their children’s behavior [24].

Numerous international studies have consistently shown that physical punishment negatively impacts child development, irrespective of its severity [25]. Key adverse outcomes identified include diminished moral internalization, increased antisocial and aggressive behaviors, both externalizing and internalizing behavior problems, lowered self-esteem, mental health issues, strained parent–child relationships, impaired cognitive and socio-emotional development, detrimental effects on brain development, and long-term consequences in adulthood such as continued antisocial behavior, mental health problems, and the perpetuation of physical punishment across generations [6,24,26,27,28,29,30,31,32,33,34,35]. Notably, no research has established any positive effects of physical punishment [26], and a broad spectrum of literature further confirms its ineffectiveness as a disciplinary measure [28,36,37,38].

Parents of young children often encounter significant challenges, given that the early years involve numerous developmental transitions within a condensed timeframe [39]. This period frequently correlates with an increased use of physical punishment [10]. Supporting this, research by Trujillo et al. [11] indicates that in Colombia, the initiation of physical punishment can occur as early as the first year of life, with a notable peak at the age of four. These findings highlight the urgent need for educational interventions targeted at parents of young children, aiming to decrease the prevalence of violence within the home and safeguard children’s development [10].

### 1.2. Parents’ Beliefs about Physical Punishment

In the context of parenting, the beliefs that parents hold significantly shape the practices they adopt to manage their children’s behavior [40]. These beliefs are defined as the “explanations, thoughts, and principles that individuals hold regarding child-rearing, guidance, and discipline” [13] (p. 30). Research across various cultural contexts indicates that a primary factor influencing parental endorsement of physical punishment is the belief in its normativity, often considered a necessary element of parenting, even for very young children [17,41,42,43]. 

Furthermore, studies have consistently found that parents’ beliefs in the positive outcomes of physical punishment are associated with its application [14,42,44,45]. Indeed, multiple studies highlight the influence of cultural values and traditions in justifying the use of corporal punishment [46]. Within this cultural framework, various opportunities emerge for the development of parental beliefs through social interactions [47]. This correlation underscores the importance of addressing and reshaping parental beliefs to reduce the use of physical punishment and highlights the importance of understanding and addressing cultural influences in shaping parental attitudes towards disciplinary practices. 

Likewise, a separate body of research indicates that parents from lower socioeconomic backgrounds are more inclined to resort to physical punishment [10,42,46], particularly when they perceive their child to be at risk or in danger [48]. Investigations into the relationship between physical punishment and early childhood have revealed contrasting beliefs. Some parents hold the view that infants are too young to comprehend right from wrong and therefore should not be subjected to physical discipline, while others believe that infants are capable of distinguishing between right and wrong and may become spoiled or defiant if not disciplined physically [49]. Another set of studies has identified parental beliefs regarding the perceived effectiveness of physical punishment in modifying child behavior and fostering long-term developmental benefits [46,50,51]. These divergent perspectives underscore the complexity surrounding parental attitudes towards physical punishment, which may be influenced by factors such as socioeconomic status and beliefs about child development.

The persistent use of corporal punishment and the belief in its necessity in some regions, despite legal prohibitions, underscore the importance of international efforts to raise awareness of legal restrictions and educate parents about alternative disciplinary methods [43]. Furthermore, research indicates that beliefs regarding the positive outcomes of physical punishment contribute to the intergenerational transmission of this parental practice [52,53]. Consequently, interventions targeting belief modification hold promise for positively impacting multiple generations [44]. 

In Barrera et al.’s meta-analysis [54] concerning brief interventions implemented in educational settings, interventions varied from a single session lasting 15 min to eight sessions each lasting 45 min. These interventions, characterized by their brevity, have exhibited enduring positive effects by targeting the modification of beliefs, thoughts, and emotions to disrupt detrimental cycles within specific social contexts. In the case of physical punishment, the objective is to intervene in the cycle of intergenerational transmission of this practice, fostering a culture of non-violence within the household.

While this approach has been previously explored in the realm of health psychology, particularly in addressing issues like tobacco use and risky sexual behavior, most studies have been confined to laboratory settings, with limited exploration of their long-term effects in natural settings [55]. Therefore, previously, a review of prior research was conducted on interventions self-identified as “brief interventions” targeting parents to decrease their utilization of violent discipline. Validated interventions were identified that varied in duration from a single 20-min session to six sessions each lasting two hours. Among these interventions, some primarily aimed to decrease parental stress, while others focused on promoting positive parenting practices [56,57,58,59,60,61,62,63,64]. One intervention, the Play Nicely program, was identified as a brief intervention that demonstrated a positive effect and specifically aimed to decrease the use of physical punishment and parents’ beliefs justifying it [65,66].

To mitigate the prevalence of physical punishment, the psychoeducational video-based program Play Nicely has been employed as a brief intervention designed to prevent child maltreatment by curbing negative parental practices, notably physical punishment. This intervention encompasses a range of disciplinary alternatives and is recognized for its efficacy, efficiency, brevity, ease of implementation, and cost-effectiveness in educating parents [65,67]. Notably, Play Nicely has been instrumental in enhancing parental confidence in their caregiving roles, with participants characterizing the program as comprehensible and considerate of familial cultural values [68].

The Play Nicely program features a comprehensive section with interactive modalities—including video, audio, and textual elements—that instruct parents on managing aggression in children aged 1 to 7 years. In empirical studies, participants were directed to engage with 4–20 selections from these offerings, with the total time invested in the intervention ranging between 5 and 10 min. The program has demonstrated substantial efficacy in diminishing the employment of physical punishment and altering parents’ perceptions and attitudes towards the purported benefits of such practices, with these effects persisting up to one year post-intervention [66,69,70]. Moreover, it has been effective in fostering the adoption of positive parenting practices [71,72,73].

### 1.3. Hypothesis

**H1.**  *Based on the preceding literature review, we hypothesize that there will be significant differences between the control group and the intervention group in the use of physical punishment, measured before and after the implementation of the Play Nicely brief intervention*.

**H2.**  *Significant differences will be observed between the control group and the intervention group in the assessment of beliefs regarding the positive effects of physical punishment, both before and after the implementation of the Play Nicely brief intervention*.

**H3.**  *A significant relationship exists between beliefs regarding the positive effects of physical punishment and its actual utilization*.

## 2. Materials and Methods

### 2.1. Design

**Scheme 1:**
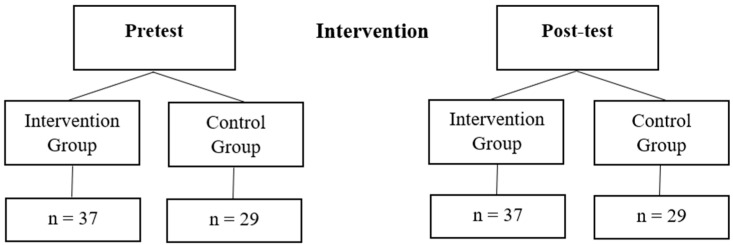
Research design.

As depicted in Scheme 1, the present study employs a quasi-experimental design with both pretest and posttest measures. The intervention group consists of 37 participants, while the control group includes 29 participants [74].

According to the study’s design, pre-test measures were administered to all participants to determine the parents’ beliefs that justify the use of physical punishment and the use of physical punishment itself. The participants were randomly assigned to either the intervention or control group, ensuring that both groups had an equal number of parents who reported using physical punishment in order to achieve homogeneity within each group. The intervention group received the Play Nicely program during a brief 15-min online session, during which they observed eight parental discipline strategies. The control group did not receive the intervention. Finally, parents from both the intervention and control groups were contacted for the post-test assessment, with an average of 41.10 days elapsed between the pre-test and post-test assessments.

### 2.2. Participants

The sample comprised 66 parents of children aged 2 to 6 years, residing in the department of Cundinamarca, Colombia. The ages of the parents ranged from 23 to 47 years, with a mean age of 35.81 years. A sociodemographic profile of the sample is detailed in Table 1.

### 2.3. Measurements

#### 2.3.1. Scale of Beliefs about the Positive Effects of Physical Punishment on Children [75]

The instrument evaluates five beliefs concerning the positive effects of physical punishment. These beliefs include: “Punishment is the best alternative for controlling children’s behavior”; “Children who have never been punished do not learn to behave appropriately”; “Children who are very aggressive should be punished to moderate their behavior”; “If punishment worked for me, it should work for my children too”; and “The stricter parents are, the better their children will be”. Each belief is rated on a scale from 1 to 10, where 1 signifies total disagreement and 10 denotes complete agreement.

#### 2.3.2. Physical Assault Scale of the Spanish Version of the Parent–Child Conflict Tactics Scale (CTSPC) [76]

The questionnaire comprises 14 items designed to assess the frequency and severity of physical punishment administered over a specified period (either the past year or month). It includes examples at various levels of severity: a minor physical punishment item is “I spanked him/her on the bottom with my hand”; a severe physical punishment item is “I pushed him/her or threw him/her to the ground”; and a very severe physical punishment item is “I burned him/her with something on purpose”. Each item is rated on a scale from 0 to 6, where 0 indicates the action never occurred, 1 indicates it occurred once, 2 twice, 3 between three and five times, 4 between six and ten times, and 5 between eleven and twenty times.

### 2.4. Procedure 

Parents were recruited through collaborations with various educational institutions in the region, which facilitated outreach via institutional email systems. Participants who responded to the invitation accessed a digital platform to review and accept the informed consent and then proceeded to complete the pretest assessment. These assessments included the belief justification for physical punishment scale and the Spanish version of the parent–child conflict tactics scale, specifically the physical assault subscale, focusing on the use of physical punishment. 

The study’s participants were randomly assigned to either the intervention or control group, ensuring that both groups had a comparable number of parents who reported employing physical punishment, thereby promoting group homogeneity. Participants were contacted via text message, formally welcomed to the study, and the online session was scheduled with parents in the intervention group. This online session, lasting approximately 15 min, included an initial greeting, a viewing of the eight selected intervention options, and a concluding farewell. Conversely, parents in the control group were simply informed of their participation in the study and were advised that they would be re-contacted for follow-up approximately one month later.

The intervention program utilized in this study was the Spanish and CD versions of the Play Nicely program developed by Vanderbilt University [65,67]. The application of the Play Nicely program in empirical studies has predominantly focused on the final section featuring 20 response options to the scenario: “Suppose you see your child hitting another child. What should you do?” Each response option is categorized as either a very good option, a good option if previous strategies have failed, or not recommended due to the availability of better alternatives.

During a single online intervention session, parents were exposed to eight pre-selected options deemed suitable for children aged 2 to 6 years, each with an approximate duration of 1 min and 15 s, resulting in a total intervention duration of 10 min. These options, delivered via audio, video, and text modalities, delineate discipline strategies for effectively addressing aggressive behavior while elucidating the reasons why negative parenting practices should be avoided for such correctional purposes. Specifically, the options presented to parents were as follows:

- Classified by the program as very good options, i.e., redirect behavior, redirect with a question, ask how the other child feels, and establish rules.
- Classified by the program as a good option if other strategies have failed: Ask your child how they feel.
- Classified by the program as not recommended because there are better alternatives: Spanking the child, ignoring the behavior, and speaking angrily.

Due to the sensitive nature of physical punishment, online administration of instruments was conducted, along with obtaining informed consent approval, to ensure participant confidentiality. The present research was conducted in accordance with the ethical principles established in the Helsinki Declaration [77] and obtained ethical approval from the Ethics Review Board of the Psychology Department at La Sabana University (Acta 083, 13 May 2015).

## 3. Results

### 3.1. Baseline Control Group and Intervention Group

In the sample, a substantial proportion of physical punishment utilization was observed, with 81.8% of participants reporting its use and 18.2% denying its employment. No participant reported utilizing very severe physical punishment, and most parents did not utilize severe physical punishment either. The items with the highest mean scores were “I spanked him/her on the buttocks with my hand” (1.65) and “I slapped his/her hand, arm, or leg” (1.015), indicating that parents employed this form of physical punishment between one and two times in the past month. 

For the intervention group, the prevalence of physical punishment utilization at baseline was 86.4%, compared to 75.8% in the control group. To assess the initial equivalence between the groups, a z-test was conducted to examine the incidence of corporal punishment between the intervention and control groups (z = 1.1101, *p* = 0.267). This analysis did not reveal any significant differences, suggesting that the samples from both the intervention and control groups are homogeneous regarding their initial use of physical punishment. 

Table 2 displays the mean scores of the sample for beliefs about the beneficial effects of physical punishment. The highest mean scores were recorded for the beliefs “the stricter parents are, the better their children will be” (3.03), “children who have never been punished do not learn to behave appropriately” (2.68), and “punishment is the best alternative for controlling children’s behavior” (2.48). Similarly, an independent sample *t*-test was conducted to determine the initial equivalence between the intervention group (IG) and control group (CG) at baseline (Table 2). The results indicated no significant differences between the two groups (*p* > 0.05), indicating that the intervention and control groups are homogeneous in their initial measure of beliefs justifying the positive effects of physical punishment.

### 3.2. Relationship between Beliefs Justifying Physical Punishment and Its Utilization

Table 3 reveals statistically significant correlations between the five beliefs about the positive effects of physical punishment and specific items from the physical assault scale of the Conflict Tactics Scale Parent–Child (CTSPC). All five beliefs were significantly correlated (*p* < 0.05) with the physical punishment item “I slapped his/her hand, arm, or leg”. Additionally, four of the beliefs demonstrated significant correlations with items 1 and 2, which involve hitting on the bottom with a hand or a hard object; the exception was the belief “The stricter parents are, the better their children will be”. The beliefs “Punishment is the best alternative for controlling children’s behavior” (*p* = 0.03) and “Children who are very aggressive should be punished to moderate their behavior” (*p* = 0.009) showed significant correlations with item 6, “I pulled his/her hair (hair pulling)”. These findings highlight that, despite low mean values in parents’ beliefs about the positive effects of physical punishment, there remains a notable use of such negative parental practices.

### 3.3. Assessment of Change in the Use of Physical Punishment

**Figure 1:**
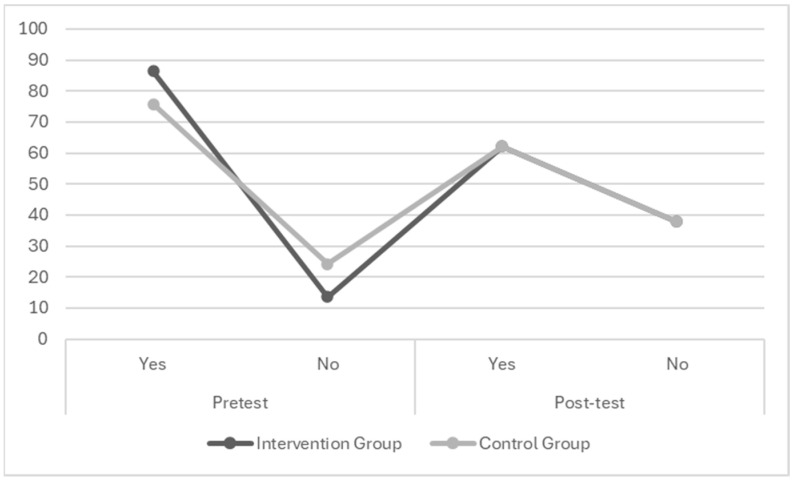
Difference in the use of physical punishment (PP) in the pretest and post-test measures of the intervention and control groups.

As depicted in Figure 1, both the intervention and control groups exhibited a reduction in the use of corporal punishment in the post-test measure. To assess the change in the utilization of physical punishment following the intervention, a z-test was conducted comparing the pretest and post-test measures. 

For the intervention group, the z-value obtained was 2.353, corresponding to a *p*-value of 0.009. This indicates a statistically significant decrease (*p* < 0.05) in the use of physical punishment between the pre- and post-intervention measures. In contrast, for the control group, the z-value was 1.065, yielding a *p*-value of 0.284. These results suggest that there are no significant differences (*p* > 0.05) between the pretest and post-test measures of physical punishment for this group.

### 3.4. Assessment of Change in Beliefs about the Positive Effects of Physical Punishment 

**Figure 2:**
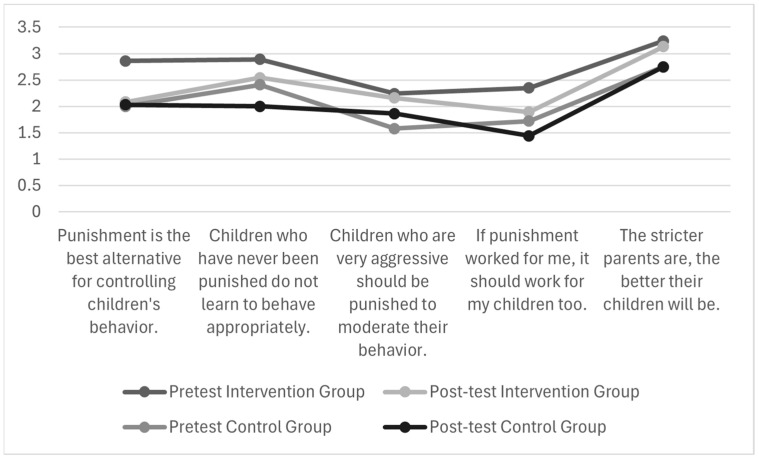
The *t*-test for related samples beliefs about the positive effects of physical punishment.

To assess the change in beliefs about the positive effects of physical punishment following the application of the brief intervention, a paired sample *t*-test was performed to compare pretest and post-test measures. In the intervention group, the results revealed a statistically significant reduction (*p* = 0.01) in the belief that “Punishment is the best alternative for controlling children’s behavior”. However, no significant differences were observed in the other beliefs measured between the pre- and post-tests. For the control group, the analysis indicated no significant changes in beliefs between the pretest and posttest measures (*p* > 0.05), as illustrated in Figure 2.

## 4. Discussion

The aim of this study was to assess the efficacy of the Play Nicely brief intervention in mitigating instances of physical punishment and the underlying beliefs that support it among parents of children aged 2 to 6 years old. The findings of the study provide evidence regarding the importance of evidence-based interventions for reducing physical punishment and the beliefs that justify them.

The results show that the prevalence of physical punishment reported by parents in the sample (81.8%) exceeded that documented in Trujillo et al.’s study [11] (77% for a Colombian sample). Moreover, given that most participants reside in municipalities within Cundinamarca, this observation aligns with the elevated prevalence identified in Hernández-Molina’s research [78] (94% for a Cundinamarca sample).

On the other hand, although the highest mean obtained in beliefs was 3.05 out of 10 for the belief “the stricter the parents, the better their children will be”, the results showed statistically significant correlations between the five beliefs assessed and the reported use of physical punishment [15,40,45]. This suggests that, although beliefs guide parenting practices [13,40], a high score in beliefs about the positive effects of physical punishment is not necessary for the presence of this negative parental practice.

In contrast to the findings of studies by Gershoff [14], Lansford et al. [16], Lansford et al. [17], and Lansford et al. [18], which indicated high levels of favorable parental beliefs towards physical punishment linked with the use of very severe physical punishment, the present research uncovered an associated pattern. It revealed that low levels of parental beliefs about the positive effects of physical punishment were correlated with the utilization of severe and low-severity corporal punishment [42]. Specifically, these beliefs demonstrated significant correlations with items associated with spanking the child on the buttocks with a hand, spanking the child on the hand, arm, or leg, and hitting the buttocks with a hard object—types of physical punishment most employed by Colombian parents, as indicated in the study by Trujillo et al. [11]. 

Regarding the primary objective of this research—to evaluate the effectiveness of the Play Nicely brief intervention in reducing parents’ reported use of corporal punishment and their beliefs about its positive effects—the intervention demonstrated efficacy. It resulted in a significant decrease in the prevalence of physical punishment utilization among participants in the intervention group compared to those in the control group [69,72]. Similarly, there was a notable reduction observed in the belief that “Physical punishment is the best alternative for controlling children’s behavior” among participants in the intervention group [66,70]. This finding is significant considering that previous studies found this belief to be one of the most prevalent across different cultures [46,50,51], including Colombia [42].

The observed effectiveness of the intervention can be attributed to the specific characteristics of the eight options presented to parents during the brief intervention session. These options provided parents with diverse strategies for correcting their children’s behavior while also offering compelling reasons why physical punishment is not a recommended approach to parenting. Moreover, the intervention focused on addressing aggressive behavior in children, a prevalent externalizing behavior that often peaks during early childhood. By delivering concrete strategies via multimedia tools, the intervention provided parents with practical guidance precisely when they needed it most—during a period marked by significant developmental challenges for their children [39,49].

However, it is noteworthy that the other beliefs did not exhibit significant decreases post-intervention. This outcome suggests, in line with existing evidence, that altering beliefs may require more time compared to modifying behavior. Consequently, while the study observed changes in the prevalence of physical punishment utilization between the pretest and posttest assessments, only one of the five beliefs regarding the positive effects of this practice exhibited a decrease. To address this, it is proposed to conduct follow-up assessments at 3 and 6 months post-intervention to monitor medium-term changes. Such follow-ups would offer valuable insights into the sustained impact of the intervention on both parental beliefs and behaviors related to physical punishment.

### 4.1. Practical Implications of the Research

Physical punishment is a negative parental practice that is commonly used in early childhood [6,7,8]. Colombia is a country with a high prevalence of this negative parental practice [10,11]. However, there are not enough validated interventions for the Colombian context. For this reason, the results of this research contribute to a brief intervention applied in the Colombian context, which demonstrated a reduction in the use of physical punishment and the beliefs that justify it. Additionally, this result contributes to the national pedagogical strategy that, based on Law 2089 [12], seeks to promote the reduction of this practice in the country [13].

Additionally, the results corroborated the correlation between the beliefs that justify physical punishment and its use [14,15,16,17,18]. This is an important focus for intervention to reduce this negative parental practice, especially in countries like Colombia that have a prolonged history of violence and armed conflict [19,20,21]. It is also important to highlight that the belief on which a significant change was observed post-intervention was “physical punishment is the best alternative to controlling children’s behavior”, highlighting the need to provide parents with alternatives to correct their children’s behavior. 

On the other hand, Play Nicely is a program that has demonstrated high long-term effectiveness in English and Spanish-speaking American parents [66,69,70,71,72,73]. However, this is the first research study in which this intervention program is applied in its Spanish version in a Latin American country, where it also demonstrated its effectiveness. Finally, this research contributes to the current focus on brief interventions characterized by their short duration, low cost, and the effects they generate by changing beliefs and interrupting cycles of violence, such as the intergenerational transmission of physical punishment [54,79].

### 4.2. Limitations of the Study and Future Research

This research, conducted with an average time lapse of approximately one month between pretest and posttest measures, would benefit from additional follow-up evaluations at 3, 6, and 12 months to ascertain the medium- and long-term effects of the intervention. Similarly, the sample size represents a limitation for the research findings; therefore, it is suggested for future research to expand it.

Moreover, while the sociodemographic variables of the sample were only examined descriptively, it is essential to acknowledge their potential influence on the low mean scores observed in the instrument assessing beliefs about the positive effects of corporal punishment. Research indicates that variables such as the number of children, parents’ age, and educational level may correlate with higher rates of physical punishment utilization and more favorable attitudes towards this practice. Therefore, future studies could benefit from exploring the relationships between these variables and parental beliefs in greater depth.

Indeed, incorporating an intervention to address parenting stress could significantly enhance the outcomes of the Play Nicely program. Parenting stress is a critical factor that influences both the frequency and intensity of corporal punishment, as stressed parents may be more likely to resort to physical measures when disciplining their children. Addressing this stress can thus play a pivotal role in mitigating aggressive parental behaviors and improving overall child-parent interactions. By integrating stress-reduction techniques and strategies into the Play Nicely program, parents may find themselves better equipped to handle challenging situations calmly and constructively without resorting to physical punishment.

## 5. Conclusions

The study allowed us to conclude that brief intervention was effective in reducing the use of physical punishment in a short period, particularly among parents with young children, who often face greater challenges in parenting. This suggests a possible lack of awareness or knowledge about alternative methods for controlling their child’s behavior. By equipping parents with alternative disciplinary strategies through the Play Nicely program, significant reductions in the use of corporal punishment were observed.

Additionally, the study revealed a noteworthy finding: despite scoring low on beliefs about the positive effects of physical punishment, parents still resort to its use. This may be due to the cultural normalization of physical punishment in parenting among Colombian parents.

The above underscores the importance of interventions that not only address parents’ immediate behaviors but also provide them with the necessary tools and resources to adopt more positive and effective parenting practices. Play Nicely serves as an example of how brief interventions can bring about significant changes in parental behavior and contribute to creating a more enriching and supportive family environment, ultimately benefiting the well-being and development of children.

As a cost-effective and time-efficient intervention, it aligns with the goal of national pedagogical strategies that encompass public policy prohibiting physical punishment in Colombia. The policy aims to reduce physical punishment and the beliefs that justify it. Despite being initially designed within a North American cultural context and previously applied mainly to Latino parents in North America, the intervention proved to be adaptable and effective for the Colombian sample.

## Figures and Tables

**Table 1:** Sociodemographic characteristics of the parents’ sample.

		Overall Sample		Intervention Group		Control Group	
Variables		F	%	F	%	F	%
Parents	Mothers	51	77.27	29	78.37	22	75.86
	Fathers	15	22.72	8	21.6	7	24.13
Marital status	Married	45	68.18	23	62.16	22	75.86
	Cohabiting/Unmarried partnership	14	21.21	9	24.32	5	17.24
	Single	6	9.09	4	10.81	2	6.89
	Separated or Divorced	1	1.51	1	2.70	0	0
Socio-economic level	High	13	19.69	6	16.21	7	24.13
	Middle	51	77.27	29	78.37	22	75.86
	Low	2	3.03	2	5.40	0	
Education	High School	5	7.57	4	10.81	1	3.44
	Technical	2	3.03	0	0	2	6.89
	Professional	22	33.33	11	29.73	11	37.93
	Postgraduate	37	56.06	22	59.45	15	51.72
Number of children	1 child	28	42.42	13	35.13	15	51.72
	2 children	30	45.45	18	48.64	12	41.37
	3 children	6	9.09	4	10.81	2	6.89
	5 children	1	1.51	1	2.70	0	
	8 children	1	1.51	1	2.70	0	

**Table 2:** Baseline beliefs about the positive effects of physical punishment in the sample.

Beliefs	Mean	Standard Deviation	Independent Sample *t*-Test (IG—CG)	Value *p*
Punishment is the best alternative for controlling children’s behavior	2.485	2.143	1.649	0.104
Children who have never been punished do not learn to behave appropriately	2.682	2.531	0.759	0.451
Children who are very aggressive should be punished to moderate their behavior	1.955	1.965	1.357	0.179
If punishment worked for me, it should work for my children too	2.076	2.093	1.213	0.230
The stricter parents are, the better their children will be	3.030	2.294	0.850	0.398

**Table 3:** Statistically significant correlations between beliefs about the positive effects of physical punishment and the utilization of this practice.

Beliefs	Type of Physical Punishment	Pearson’s r
Punishment is the best alternative for controlling children’s behavior.	2. I spanked him/her on the bottom with something like a belt, brush, stick, or another hard object.	0.412 ***
	3. I slapped his/her hand, arm, or leg.	0.291 *
	6. I pulled his/her hair (hair pulling).	0.263 *
Children who have never been punished do not learn to behave appropriately.	1. I spanked him/her on the bottom with my hand.	0.352 **
	2. I spanked him/her on the bottom with something like a belt, brush, stick, or another hard object.	0.322 **
	3. I slapped his/her hand, arm, or leg.	0.245 *
Children who are very aggressive should be punished to moderate their behavior.	1. I spanked him/her on the bottom with my hand.	0.415 ***
	2. I spanked him/her on the bottom with something like a belt, brush, stick, or another hard object.	0.314 *
	3. I slapped his/her hand, arm, or leg.	0.258 *
	6. I pulled his/her hair (hair pulling).	0.321 **
If punishment worked for me, it should work for my children too.	1. I spanked him/her on the bottom with my hand.	0.410 ***
	2. I spanked him/her on the bottom with something like a belt, brush, stick, or another hard object.	0.275 *
	3. I slapped his/her hand, arm, or leg.	0.312 *
The stricter parents are, the better their children will be.	1. I spanked him/her on the bottom with my hand.	0.525 ***
	3. I slapped his/her hand, arm, or leg.	0.285 *

* *p* < 0.05; ** *p* < 0.01; *** *p* < 0.001.

## Data Availability

Our research data are available upon request to interested researchers. The above is because the database contains sensitive information about the use of physical punishment in families. Additionally, given the size of the sample and the data contained therein, it could facilitate the identification of participants. For this reason, the confidentiality of the participants is sought to be ensured.
